# Esophageal Atresia With or Without Tracheoesophageal Fistula: Comorbidities, Genetic Evaluations, and Neonatal Outcomes

**DOI:** 10.7759/cureus.34779

**Published:** 2023-02-08

**Authors:** Divya Khattar, Kristen R Suhrie

**Affiliations:** 1 Department of Neonatal Perinatal Medicine, Cincinnati Children's Hospital Medical Center, Cincinnati, USA; 2 Department of Pediatrics and Medical and Molecular Genetics, Indiana University School of Medicine, Indianapolis, USA

**Keywords:** whole-genome sequencing, whole-exome sequencing, genetic testing, birth defects, congenital anomalies, esophageal atresia, tracheoesophageal fistula

## Abstract

Objective

Esophageal atresia with or without tracheoesophageal fistula (EA/TEF) has a reported incidence of 1 in 3500 live births and requires intensive care and surgery. To evaluate the prevalence of a molecularly confirmed genetic etiology of EA/TEF in a level IV neonatal intensive care unit (NICU), focusing on genetic evaluation, diagnostic yield, and clinical outcomes of these neonates.

Study design

A retrospective cohort study over a period of seven years was performed for all patients admitted with a diagnosis of EA/TEF. Automated data was extracted for demographic information and manual extraction was done to evaluate the frequency of associated anomalies, type of genetic evaluations and diagnoses, and outcomes at NICU discharge.

Results

Sixty-eight infants met the inclusion criteria. The majority were male (n=42; 62%), born at >37 weeks' gestation (n=36; 53%), and had EA with distal TEF (n=54; 79%). Most (n=53; 78%) had additional associated congenital anomalies, but only 47 (69%) patients had a genetics evaluation performed and genetic testing was sent for 44 (65%) of those patients. The most common genetic testing performed was chromosomal microarray analysis (n=40; 59%), followed by chromosome analysis (n=11; 16%), and whole exome/genome sequencing (n=7; 10%). Five unique genetic diagnoses including CHARGE Syndrome, Fanconi Syndrome, EFTUD2-related mandibulofacial dysostosis, and two different chromosomal deletion syndromes were made for a total of nine (13%) patients in our cohort. The cohort suffered a high rate of morbidity and mortality during their NICU stay with important differences noted in isolated vs non-isolated EA/TEF. Twelve infants (18%) died prior to NICU discharge. Of those surviving, 40 (71%) infants had a primary repair, 37 (66%) infants required G or GJ feedings at NICU discharge, and eight (14%) patients were discharged on some type of respiratory support.

Conclusion

In this high-risk cohort of EA/TEF patients cared for at a quaternary NICU, a majority were non-isolated and had some form of a genetic evaluation, but a minority underwent exome or genome sequencing. Given the high prevalence of associated anomalies, high mortality, and genetic disease prevalence in this cohort, we recommend standardization of phenotyping and genetic evaluation to allow for precision care and appropriate risk stratification.

## Introduction

Esophageal atresia (EA) with or without tracheoesophageal fistula (TEF) is a congenital developmental defect of the gastrointestinal tract with a prevalence of nearly 2.3 per 10,000 live births [1]. There are certain non-specific signs on prenatal ultrasound including polyhydramnios, small fetal stomach, and dilated esophageal pouch that can lead to suspicion for EA/TEF, but prenatal detection remains challenging with a low overall detection rate [2,3]. Patients identified after birth can present with copious oral secretions, gagging, and/or respiratory distress in the neonatal period [4]. There are five anatomical subtypes (A through E) of EA/TEF as per the Gross classification based on the presence and location of TEF, with subtype C (EA with distal TEF) being the most common type [5].

Early tracheoesophageal development is determined by a time-dependent and localized expression of various growth factors (FGF4), transcription factors (SOX2, FOXA2), and molecular pathways, such as the sonic hedgehog, Hox, and retinoic acid pathways [6,7]. Disruption in this development results in EA/TEF, which can occur as an isolated defect or in conjunction with additional congenital anomalies [8], and may involve almost any other organ system including cardiac, musculoskeletal, genitourinary, and neurological [9]. The underlying mechanism leading to EA/TEF largely remains unknown, but numerous genetic pathways and environmental susceptibility factors have been identified [10]. Multiple studies in the literature have identified a variety of genetic mechanisms such as aneuploidies (trisomy 18), chromosomal microdeletion or microduplication syndromes (22q11.2 deletion syndrome), and a variety of single gene disorders (CHD7, SOX2, FANCB, MYCN, TCF4, NRXN1) that lead to the development of EA/TEF [11,12]. Among environmental factors, antenatal exposure to methimazole, alcohol, cigarette smoke, diethylstilbestrol (DES), and maternal diabetes have been implicated but definitive associations remain unproven [10]. 

There are very few retrospective studies [2,13] or case reports [14,15] describing what genetic testing is helpful in this population or the types and numbers of genetic diagnoses made from that testing. A retrospective study by Beauregard-Lacroix et al. [13] in a cohort of complex EA (n= 45) looked at types of genetic testing which included chromosome analysis (73%), chromosomal microarray analysis (31%) and diepoxybutane for Fanconi anemia (31%) which yielded seven molecular diagnoses in a total of 33 patients tested. In another cohort in France [16] of 116 patients with EA, the genetic yield was around 12%; however, the type and yield of genetic testing were not reported. A more recent study looking at genetic diagnoses in fetuses prenatally diagnosed with EA [2] had a yield of seven molecular diagnoses in a cohort of 61 patients. Nearly half of this cohort had prenatal genetic testing performed (chromosome analysis, chromosomal microarray analysis, or FISH for aneuploidies) and 18 additional infants had postnatal genetic testing which also comprised of microarray (57%), chromosome analysis (37%), or single gene (27%). Furthermore, many genetic disorders have phenotypes that overlap with VACTERL association which includes chromosomal deletion/duplication syndromes [15] and single gene disorders [17]. A larger study by Guptha et al. including 1,175 cases of EA/TEF showed that less than 25% of patients meet the diagnostic criteria for VACTERL association suggesting the importance of genetic evaluation in this cohort [18].

A recent large multicenter study characterized mortality rates and hospital lengths of stay (LOS) in neonates with EA/TEF based on birth weight and congenital anomalies [19]. A better understanding of any comorbid anomalies and/or genetic conditions in EA/TEF patients can be used prenatally to manage pregnancies, counsel families more effectively on recurrence risk and prognosis, and facilitate an appropriate delivery plan. Postnatally, this information can help with surgical planning, risk stratification, and resource planning. The goal of this retrospective cohort study was to evaluate the frequency and type of testing used to fully phenotype a patient with EA/TEF, associated anomalies identified, genetic testing performed, and the prevalence of a molecularly confirmed genetic diagnosis in patients cared for at a single-level IV neonatal intensive care unit (NICU). NICU outcomes were then stratified by the presence or absence of associated anomalies.

## Materials and methods

A retrospective cohort study was completed for all patients with a diagnosis of esophageal atresia (EA) and/or tracheoesophageal fistula (TEF) who were admitted to a level IV NICU from January 1, 2013 - December 31, 2019. Patients with only a suspicion for EA/TEF but confirmatory clinical evaluation suggestive of an alternate diagnosis were excluded. This study was approved by the CCHMC Institutional Review Board (Study Number 2020-0389) with a waiver of parental consent.

Automated data were extracted from the patient’s electronic medical record (EMR) for demographic information and NICU admission and discharge dates. All the charts were then reviewed manually to extract the following data - prenatal diagnosis status, type of TEF, associated malformations including rates of test utilization such as echocardiography, renal ultrasound, medical genetics consultation, genetic testing completed and associated results, the presence of a genetic diagnosis, and mortality prior to NICU discharge. Additionally, we looked at infants’ evaluation and testing for VACTERL association and if a diagnosis of VACTERL was noted. We classified our cohort into ‘isolated’ and ‘non-isolated’ EA/TEF (those with any additional anomalies) and compared clinical outcomes in those surviving discharge for the type of repair, feeding, and airway support at the time of discharge, and length of stay in the NICU. Being a retrospective study, data collection was limited to medical record review and subject to selection bias as more complex cases were likely to be referred to our national referral center. To mitigate bias, we included isolated EA/TEF patients and attempted to review outside hospital records prior to transfer, when available. 

Demographic variables were explored using appropriate descriptive statistics. Continuous data are presented as medians with standard deviation (SD) and categorical data are presented as frequencies with percentages. Statistical analysis was performed using Fisher’s exact test for categorical variables and the Mann-Whitney test for continuous variables using GraphPad Prism 9.3.1 (350) for MacOS (GraphPad Software, San Diego, CA). A p-value of 0.05 was considered the threshold for statistical significance.

## Results

Demographics

A total of 68 infants met the inclusion criteria; Table 1 demonstrates the characteristics of the cohort. The mean gestational age in our cohort was 35.57 weeks (SD ± 3.65 weeks) and the mean birth weight was 2.37 kg (SD ± 0.83 grams). The most common type of TEF in our cohort was EA with distal TEF (Type C, n=54; 79%), followed by EA without TEF (Type A, n=6; 9%), H-type TEF (Type E, n=6; 9%), and EA with proximal TEF (Type B, n=2; 3%). Of the 26 patients that had a prenatal suspicion of EA, most were identified based on prenatal ultrasound findings and only five patients had an advanced fetal MRI imaging performed.

**Table 1:** Cohort demographic data EA: esophageal atresia; EA/TEF: esophageal atresia with or without tracheoesophageal fistula; NICU: neonatal intensive care unit; SVD: spontaneous vaginal delivery

		N (%)
Sex	Male	42 (62%)
	Female	26 (38%)
Race	White	56 (82%)
	Black	3 (4%)
	Other	9 (13%)
Prenatal suspicion of EA	Yes	26 (38%)
	No	42 (62%)
Gestational age	37 weeks	36 (53%)
	< 37 weeks	32 (47%)
Birth weight	2500 gm	34 (50%)
	< 2500 gm	34 (50%)
Mode of delivery	SVD	29 (43%)
	C-Section	39 (57%)
Isolated EA/TEF	Yes	15 (22%)
	No	53 (78%)
NICU mortality	Yes	12 (18%)
	No	56 (82%)

Associated anomalies

The majority (n=53; 78%) had additional associated congenital anomalies. Table 2 classifies those anomalies by organ system and describes the specific anomalies seen in the cohort as well as the incidence of each organ system anomaly across the cohort. In order to fully characterize the phenotype, these infants underwent further imaging (ECHO, renal or spinal ultrasound, brain MRI) and/or evaluation by various specialists. We determined what proportion of the cohort underwent a complete radiology evaluation which included a renal ultrasound, spinal X-rays, and an echocardiogram. All infants had spinal X-rays and echocardiography performed, and 59 (87%) infants had a renal ultrasound completed. Of the 53 patients that had additional anomalies, 15 (28%) infants had one additional anomaly and 38 (72%) infants had two or more additional anomalies.

**Table 2:** Associated congenital anomalies in non-isolated TEF/EA EA/TEF: esophageal atresia with or without tracheoesophageal fistula; VSD: ventricular septal defect; ASD: atrial septal defect

Associated Anomalies	N (%)
Musculoskeletal (vertebral and rib anomalies, enchondroma, congenital kyphosis/scoliosis, bifid or absent thumb)	16 (23%)
Neurological (Craniosynostosis, microcephaly, tethered cord)	9 (14%)
Ophthalmological (microphthalmia, coloboma, esotropia, exotropia)	5 (7%)
Ear/nose/airway including respiratory system (except EA/TEF) (agenesis or hypoplastic lung, tracheal stenosis, tracheal rings, cleft palate, choanal atresia)	12 (18%)
Congenital heart defects (VSD, bicuspid aortic valve, hypoplasia of aortic arch, secundum ASD, pulmonary atresia)	26 (38%)
Gastrointestinal (Hirschsprung’s disease, imperforate anus, duodenal atresia, omphalocele)	14 (20%)
Urinary system (congenital hydronephrosis, duplex or ectopic kidney, renal agenesis)	13 (19%)
Genital system (Chordee, hypospadias)	4 (6%)
Dermatologic (hemangioma, nevus)	5 (7%)

Nearly 30% of the patients had a differential diagnosis for VACTERL association. In our cohort, all of the patients underwent both a complete physical exam and radiological evaluation, only 13 (19%) met the criteria for a diagnosis of VACTERL association; and 11 (85%) had some sort of genetic testing sent with no diagnostic yield.

Genetic evaluation and testing

**Figure 1:**
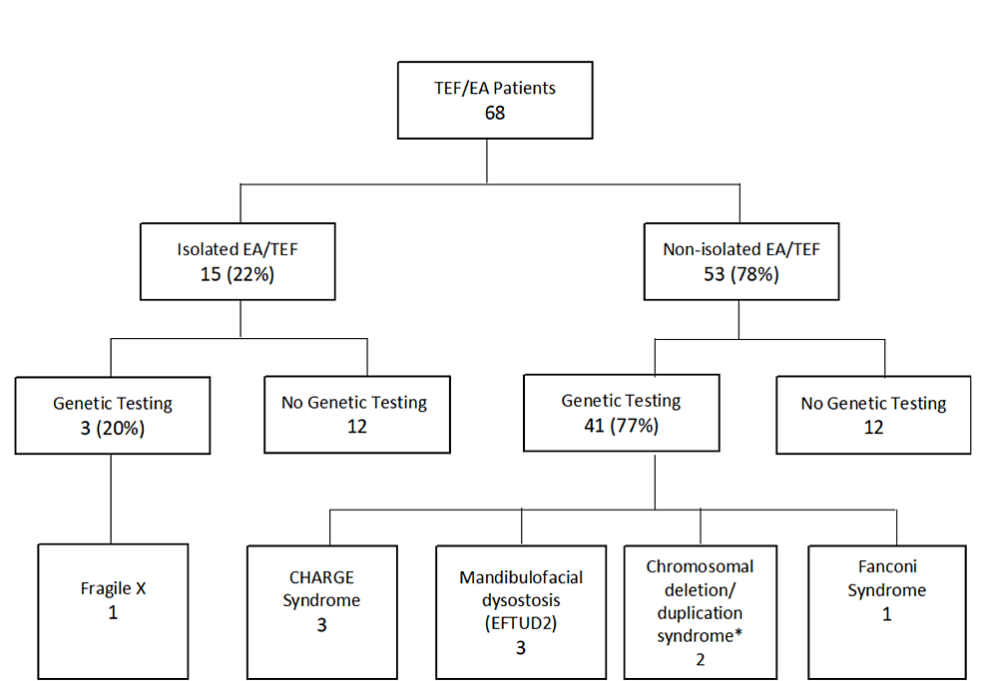
Genetic testing and subsequent diagnoses for patients with isolated and non-isolated EA/TEF * 7q11.2 chromosomal deletion syndrome with partial AUTS2 gene deletion and 3q26.32-3q27.1 deletion syndrome including SOX2 gene deletion. EA/TEF: esophageal atresia with or without tracheoesophageal fistula; CHARGE Syndrome: coloboma, heart defects, atresia choanae, growth retardation, genital abnormalities, and ear abnormalities

**Figure 2:**
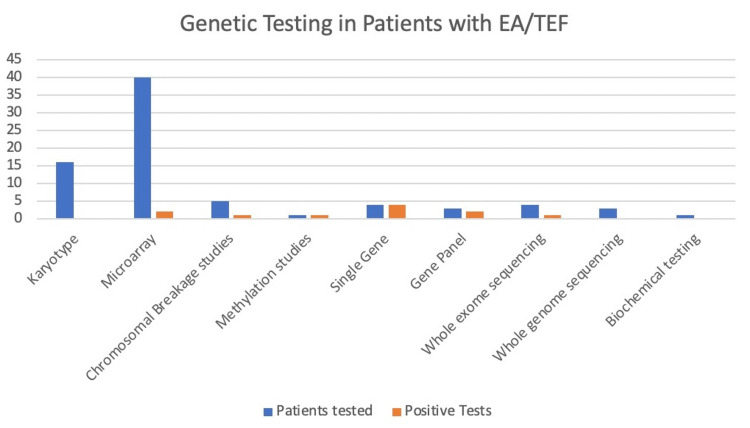
Types of genetic testing and associated diagnostic yields

Medical genetic consultations and evaluations were performed for 47 (69%) patients with some type of genetic testing performed for 44 of those patients. For a majority of the patients (n=43; 91%), the initial genetic evaluations occurred during their NICU stay. Five patients (7%) had prenatal testing consisting of chromosome analysis and chromosomal microarray analysis, all of which were non-diagnostic. Figure 1 shows the proportion of patients that underwent a combination of prenatal and postnatal genetic testing stratified by isolated or non-isolated types of EA/TEF with the resulting number and types of diagnoses. The types and yield of the various genetic tests performed on the patient cohort are illustrated in Figure 2. The most common genetic test performed was a chromosomal microarray analysis, which was done in 40 patients, which was 91% of those who underwent some form of genetic testing, and 59% of the entire cohort. Chromosomal microarray analysis testing was positive for a genetic diagnosis in two (5%) patients that underwent testing. Additional testing patterns and diagnostic yields are highlighted in Figure 2. In total, a molecular genetic diagnosis associated with the phenotype of EA/TEF was confirmed in nine (13%) study participants. All of these patients had non-isolated EA/TEF with additional associated congenital anomalies. An unrelated genetic diagnosis of Fragile X syndrome was made in the outpatient department in a patient with an isolated EA/TEF which we did not include in our total diagnostic yield for this cohort. The nine genetic diagnoses found in the cohort are listed at the bottom of Figure 1 and their phenotypic characteristics are described in Table 3.

**Table 3:** Phenotypic data for genetic diagnoses EA: esophageal atresia; TEF: tracheoesophageal fistula; VSD: ventricular septum defect; AV canal defect: atrio-ventricular canal defect; CHARGE Syndrome: coloboma, heart defects, atresia choanae, growth retardation, genital abnormalities, and ear abnormalities

Patient Number	Phenotype	Genetic Diagnoses	Gene	Inheritance
1	EA with distal TEF, microcephaly, micrognathia, hyperopia and astigmatism, microtia	Mandibulofacial dysostosis with microcephaly	EFTUD2 (NM_004247.3:c.1058+1G>A)	Autosomal dominant
2	EA with distal TEF, Bilateral choanal atresia, right eye coloboma, micropenis	CHARGE Syndrome	CHD7 (NM_017780.4:c.1480C>T)	Autosomal dominant
3	EA with distal TEF, micrognathia, cleft palate, microcephaly, accessory ear tags	Mandibulofacial dysostosis with microcephaly	EFTUD2 (NM_004247.3:c.969del)	Autosomal dominant
4	EA with distal TEF, secundum atrial septal defect	Interstitial deletion of 230.8 kb from 7q11.22 of clinical significance	7q11.22(69564501_69795311) x1, hg19 including partial AUTS2 gene deletion	Autosomal dominant
5	EA with distal TEF, congenital bicuspid aortic valve, absent bilateral thumbs, ectopic left kidney, esotropia, hyperopic astigmatism	Fanconi Anemia	Pathogenic homozygous deletion of FANCA chr16:89851162-89851471, hg19	Autosomal recessive
6	EA with distal TEF, mitral valve stenosis, cleft palate, left ear microtia, right preauricular tag, exotropia, amblyopia, astigmatism, mild left kidney pelviectasis	Mandibulofacial dysostosis with microcephaly syndrome	EFTUD2 (NM_001258353:c.969del)	Autosomal dominant
7	EA with distal TEF, bilateral choanal atresia, right anophthalmia, left microphthalmia, aqueductal stenosis, Rhombencephalosynapsis,	Interstitial deletion of 5.1 Mb from 3q26.32- 3q27.1 of clinical significance	3q26.32q27.1(178333277_183386204) x1, hg19 including SOX2 gene deletion	Autosomal dominant
8	EA with distal TEF, aberrant right subclavian artery, VSD, right coloboma, tracheobronchomalacia, rib synostosis	CHARGE Syndrome	CHD7 (NM_017780.4:c.4393C>T)	Autosomal dominant
9	EA with distal TEF, AV canal defect, thymic hypoplasia, bicuspid aortic valve, right aortic arch with left subclavian artery, bilateral congenital choanal atresia, horseshoe kidney, ectrodactyly of both hands, bilateral colobomas, club foot	CHARGE Syndrome	CHD7 (NM_017780.4:c.4480C>T)	Autosomal dominant

Clinical outcomes

Table 4 describes outcomes at NICU discharge stratified by isolated or non-isolated EA/TEF. A majority of those surviving hospital discharge underwent an early primary repair of EA/TEF (n=40; 71%) and needed artificial feeding support at the time of NICU discharge (NG, NJ, G tube, or GJ tube, (n=43; 77%)), and were discharged without respiratory support (n=48; 86%). Twelve patients, 18% of the cohort, died during their NICU admission; most (n=8; 67%) had undergone EA/TEF repair.

**Table 4:** Clinical outcomes in TEF/EA patients at NICU discharge ^*p < 0.05^ ^a ^Three tracheostomy patients were ventilator-dependent at discharge ^b^ Comparing PO feeds to any type of tube feedings ^c^ Comparing room air to any type of respiratory support EA/TEF: esophageal atresia with or without tracheoesophageal fistula; PO: per os/by mouth; NG/NJ; naso-gastric/naso-jejunal; G tube/GJ: gastrostomy tube/gastro-jejunal

Outcomes		Isolated n=14(%)	Non-isolated n=42(%)	p-value
Type of repair	Early Primary	12 (86)	28 (67)	0.31
	Delayed Primary	2 (14)	14 (33)	
Feeding support	PO	7 (50)	6 (14)	0.01*^b^
	NG/NJ	1 (7)	5 (12)	
	G tube/GJ	6 (43)	31 (74)	
Respiratory support	Room Air	14 (100)	34 (81)	0.18^c^
	Nasal cannula	0 (0)	3 (7)	
	Tracheostomy	0 (0)	5^a^ (12)	
Length of stay (days) median (range)		46 (11-128)	92 (14-624)	0.028*
Mortality		2 (14)	10 (71)	0.71

## Discussion

In our retrospective cohort study of postnatally confirmed EA/TEF receiving care at a level IV NICU, the majority, 78%, had additional anomalies, however, only 69% of patients were evaluated by a geneticist. Some form of genetic testing was sent for 65% of patients with 13% receiving a primary genetic diagnosis; 20% of those tested. Patients with non-isolated EA/TEF were more likely to undergo genetic testing (77% vs 20%, p-value < 0.05), and not all patients that underwent genetic testing received the same type(s) of genetic testing. A majority of patients underwent complete phenotyping to identify other anomalies associated with EA/TEF. Multiple studies [16,20] have demonstrated associated anomalies in cardiovascular, urogenital, and gastrointestinal systems, highlighting the need for thorough clinical evaluation in these patients, and this study also demonstrated that when comprehensive phenotyping is undertaken, a majority, 78%, are non-isolated. Of note, during the study period, there were no standards in place, either national or institutional practice guidelines, as to what medical evaluations such as echocardiography or renal ultrasound, should be performed in patients with EA/TEF or what genetic testing if any, should be undertaken. Often, patients with EA/TEF are assigned a diagnosis of VACTERL without meeting all criteria or having undergone any genetic testing as seen in our cohort and others [2]. Guptha et al. described that EA/TEF is more likely to be a part of an alternate syndrome other than VACTERL using a large registry [18] and thus requires comprehensive genetic testing evaluation even when a diagnosis of VACTERL is in the differential diagnosis [2].

The genetic diagnoses in our cohort (CHARGE Syndrome, Fanconi Syndrome, EFTUD2-related mandibulofacial dysostosis) have been reported in the literature to be associated with EA/TEF [4,12]. The diagnosis with an interstitial deletion of 230.8 kb from the long arm of chromosome 7 (7q11.22 which includes partial AUTS2 gene deletion) has been associated with AUTS2 syndrome with intellectual disability, speech, and language delays, dysmorphic facies, and some rare congenital malformations [21] although EA/TEF has not been reported in the literature with this deletion. The other genetic diagnosis included an interstitial deletion of 5.1 MB from 3q26.32-3q27.1 of clinical significance which included the SOX2 gene. Patients with this deletion present with growth restriction, feeding difficulties, intellectual disability, speech delay, dysmorphic facies, hypotonia, and pubertal delay/genital abnormalities. This deleted region includes SOX2 which is associated with syndromic microphthalmia-3, also known as SOX2 anophthalmia syndrome characterized by brain and ophthalmological abnormalities, seizures, as well as EA/TEF [22,23].

Other common genetic etiologies associated with EA/TEF include chromosomal disorders (trisomy 21, trisomy 18, trisomy 13), Feingold syndrome (MYCN), Fanconi anemia (multiple genes including FANCB, FANCC, FANCG, BRIP1) and other recurrent deletion and duplication syndromes [4,11]. Of note, the most common genetic mechanism, either in our cohort or in the other published studies of EA/TEF patients, involves single gene variants. The most common genetic test sent in our cohort was a chromosomal microarray analysis (n = 40), followed by chromosome analysis (n = 16); and only seven patients had an evaluation with whole exome/genome sequencing (ES/GS). A recent study by Sy et al. evaluated clinical exome sequencing results in 67 individuals with EA/TEF+ (EA/TEF associated with other major birth defects) and found a definitive or probable diagnosis in 11/67 (16%). They also described candidate genes like TCF4 and NRXN1 as having sufficient evidence to support an association with EA/TEF and others like NSD1, PTPN11, and FLNA with insufficient evidence to support association [12]. Another large study by Zhong et al. performed whole genome sequencing on 185 trios that included 59 isolated and 126 complex cases and found a significant burden of protein-altering de-novo coding variants in the complex cases although many were classified as VUS [24]. They concluded that the number of risk genes contributing is very large, highlighting the genetic heterogeneity of EA/TEF. More recently, an evidence-based clinical guideline published by the American College of Medical Genetics and Genomics (ACMG) in 2021 strongly recommended ES/GS sequencing as the first- or second-tier testing for patients with congenital anomalies [25]. It is important to note that early and timely genetic testing with rapid ES/GS in the NICU can significantly increase diagnostic yields in patients with major birth defects allowing for precision care and appropriate counseling for families about short and long-term outcomes as well as recurrence risk. Various studies have looked at the impact of ES/GS in the NICU for critically ill infants with suspected genetic disorders and found a faster and higher diagnostic yield resulting in a change in clinical management, reduction in infant morbidity and mortality as well as overall cost savings [26-28]. Caring for patients with EA/TEF is labor-intensive and complex. After experiencing an initial NICU stay that lasts well over two months for a majority of patients, most require artificial feeding support at discharge and need ongoing specialized outpatient care. Important differences were seen in those with isolated vs non-isolated EA/TEF which included a higher rate of artificial feeding support at discharge as well as a length of stay that was twice as long. It is important to incorporate these findings into parental counseling, be it prenatally or shortly after NICU admission once a full phenotypic and genotypic picture has been obtained.

We acknowledge that retrospective studies such as this are intrinsically biased due to limitations in electronic health records and a rapidly changing genetic testing landscape over the years. However, for rare diseases, it provides insight into significant knowledge gaps to improve care and can be used for quality improvement initiatives and prospective studies. There is significant genetic heterogeneity in addition to possible multifactorial etiology of EA/TEF which highlights the need for a standardized, unbiased approach to genomic care for this population. We recognize that there is still much to be understood about the genetic mechanisms of EA/TEF and that even a non-diagnostic ES/GS does not rule out a genetic cause for the patient's symptoms but does provide a data set that can be reinterrogated in the future as we gain further insight into the genetic mechanisms of disease. Additionally, ES/GS can identify variants of uncertain significance (VUS) in disease-causing as well as candidate genes that can be difficult to interpret and use clinically. This signifies the importance of sustained research efforts to identify risk genes for EA/TEF in humans.

## Conclusions

In conclusion, infants with EA/TEF need a detailed determination of their complete phenotype. This starts with a physical exam which includes special attention to limb, ophthalmological, genital, and anal development, facial morphology, and a review of growth parameters. All patients also require an echocardiogram, renal ultrasound, and spinal X-rays to evaluate for cardiac, renal, and vertebral anomalies respectively. When vertebral anomalies are noted, further spinal imaging with either ultrasound or MRI is needed to evaluate for spinal cord abnormalities. Often, infants with EA/TEF have additional anomalies identified in their NICU hospitalization which places them at a higher risk of having an underlying genetic etiology. Along with thorough clinical phenotyping, we strongly recommend that a medical genetics consultation be performed for all patients with EA/TEF in the NICU and for that relationship to continue as they transition to the outpatient setting. Reviewing our own cohort and other recent studies, we recognize a recurrent pattern of monogenic disorders (like CHARGE Syndrome, Fanconi anemia, Feingold syndrome, and other microdeletion and duplication syndromes) that have a distinct phenotype albeit challenging to recognize in the NICU. Based on the literature summarized above, for EA/TEF patients, a rapid ES/GS would be the most effective genetic test in the NICU to get a timely diagnosis. However, this testing is not widely available and may be cost-prohibitive at some institutions and hospital systems. In those situations, and when a distinct phenotype is not recognizable in these patients, we would recommend starting with a microarray and a gene panel that covers the genes already associated with EA/TEF (including CHD7, SOX2, EFTUD2, MYCN, FANCA, FANCB, FANCC, FGFR3, NRXN1, TCF4) with the possibility to reflex to ES/GS if initial findings are inconclusive. By fully characterizing the phenotype and genotype of a patient with EA/TEF in the NICU, a comprehensive care plan can be developed, and parents can be given a better picture of their child’s healthcare needs and expected outcomes surrounding nutritional and medical milestones. Further work needs to be done to fully characterize the genetic etiology of this complex birth defect to better understand genotype-phenotype relationships and predict long-term outcomes.

## References

[1] Lupo PJ, Isenburg JL, Salemi JL (2017). Population-based birth defects data in the United States, 2010-2014: A focus on gastrointestinal defects. Birth Defects Res.

[2] Rohanizadegan M, Tracy S, Galarreta CI (2020). Genetic diagnoses and associated anomalies in fetuses prenatally diagnosed with esophageal atresia. Am J Med Genet A.

[3] Pardy C, D'Antonio F, Khalil A, Giuliani S (2019). Prenatal detection of esophageal atresia: A systematic review and meta-analysis. Acta Obstet Gynecol Scand.

[4] Scott DA (2009). Esophageal atresia / tracheoesophageal fistula overview. Gene Reviews.

[5] Gross RE, Ladd WE (1953). The Surgery of Infancy and Childhood: Its Principles and Techniques. British Journal of Surgery.

[6] Solomon BD (2011). VACTERL/VATER Association. Orphanet J Rare Dis.

[7] Brosens E, Marsch F, de Jong EM (2016). Copy number variations in 375 patients with oesophageal atresia and/or tracheoesophageal fistula. Eur J Hum Genet.

[8] Shaw-Smith C (2006). Oesophageal atresia, tracheo-oesophageal fistula, and the VACTERL association: review of genetics and epidemiology. J Med Genet.

[9] Davari HA, Hosseinpour M, Nasiri GM, Kiani G (2012). Mortality in esophageal atresia: Assessment of probable risk factors (10 years’ experience). J Res Med Sci.

[10] de Jong EM, Felix JF, de Klein A, Tibboel D (2010). Etiology of esophageal atresia and tracheoesophageal fistula: "mind the gap". Curr Gastroenterol Rep.

[11] Felix JF, de Jong EM, Torfs CP, de Klein A, Rottier RJ, Tibboel D (2009). Genetic and environmental factors in the etiology of esophageal atresia and/or tracheoesophageal fistula: an overview of the current concepts. Birth Defects Res A Clin Mol Teratol.

[12] Sy MR, Chauhan J, Prescott K (2022). Exome sequencing efficacy and phenotypic expansions involving esophageal atresia/tracheoesophageal fistula plus. Am J Med Genet A.

[13] Beauregard-Lacroix E, Tardif J, Lemyre E, Kibar Z, Faure C, Campeau PM (2017). Genetic testing in a cohort of complex esophageal atresia. Mol Syndromol.

[14] Klaniewska M, Toczewski K, Rozensztrauch A (2021). Occurrence of esophageal atresia with tracheoesophageal fistula in siblings from three-generation family affected by variable expressivity mycn mutation: a case report. Front Pediatr.

[15] Nguyen LT, Fleishman R, Flynn E (2017). 22q11.2 microduplication syndrome with associated esophageal atresia/tracheo-esophageal fistula and vascular ring. Clin Case Rep.

[16] Stoll C, Alembik Y, Dott B, Roth MP (2017). Associated anomalies in cases with esophageal atresia. Am J Med Genet A.

[17] Chen Y, Liu Z, Chen J (2016). The genetic landscape and clinical implications of vertebral anomalies in VACTERL association. J Med Genet.

[18] Guptha S, Shumate C, Scheuerle AE (2019). Likelihood of meeting defined VATER/VACTERL phenotype in infants with esophageal atresia with or without tracheoesophageal fistula. Am J Med Genet A.

[19] Keefe G, Culbreath K, Edwards EM (2022). Current outcomes of infants with esophageal atresia and tracheoesophageal fistula: A multicenter analysis. J Pediatr Surg.

[20] Galarreta CI, Vaida F, Bird LM (2020). Patterns of malformation associated with esophageal atresia/tracheoesophageal fistula: A retrospective single center study. Am J Med Genet A.

[21] Sanchez-Jimeno C, Blanco-Kelly F, López-Grondona F (2021). Attention deficit hyperactivity and autism spectrum disorders as the core symptoms of AUTS2 syndrome: description of five new patients and update of the frequency of manifestations and genotype-phenotype correlation. Genes (Basel).

[22] Williamson KA, Hever AM, Rainger J (2006). Mutations in SOX2 cause anophthalmia-esophageal-genital (AEG) syndrome. Hum Mol Genet.

[23] Williamson KA, Yates TM, FitzPatrick DR (2006). SOX2 disorder. GeneReviews.

[24] Zhong G, Ahimaz P, Edwards NA (2022). Identification and validation of candidate risk genes in endocytic vesicular trafficking associated with esophageal atresia and tracheoesophageal fistulas. HGG Adv.

[25] Manickam K, McClain MR, Demmer LA (2021). Exome and genome sequencing for pediatric patients with congenital anomalies or intellectual disability: an evidence-based clinical guideline of the American College of Medical Genetics and Genomics (ACMG). Genet Med.

[26] Petrikin JE, Cakici JA, Clark MM (2018). The NSIGHT1-randomized controlled trial: rapid whole-genome sequencing for accelerated etiologic diagnosis in critically ill infants. NPJ Genom Med.

[27] Meng L, Pammi M, Saronwala A (2017). Use of exome sequencing for infants in intensive care units: ascertainment of severe single-gene disorders and effect on medical management. JAMA Pediatr.

[28] Farnaes L, Hildreth A, Sweeney NM (2018). Rapid whole-genome sequencing decreases infant morbidity and cost of hospitalization. NPJ Genom Med.

